# Supplementary material for: Bayesian refinement of protein structures and ensembles against SAXS data using molecular dynamics
Source: PLoS Comput Biol. 2017 Oct 18;13(10):e1005800. doi: 10.1371/journal.pcbi.1005800 (PMC5662244; doi:10.1371/journal.pcbi.1005800)
Supplement: S1 Archive — (BZ2) [file pcbi.1005800.s011.tar.bz2 › gromacs_4.62_bayesian_saxs_refi/share/html/online/g_density.html]

g\_density


| g\_density Main Table of Contents |

**VERSION 4.6  
Sat 19 Jan 2013**

---

### Description

Compute partial densities across the box, using an index file.

For the total density of NPT simulations, use g\_energy instead.

Densities are in kg/m^3, and number densities or electron densities can also be
calculated. For electron densities, a file describing the number of
electrons for each type of atom should be provided using -ei.
It should look like:  
2  
atomname = nrelectrons  
atomname = nrelectrons  
The first line contains the number of lines to read from the file.
There should be one line for each unique atom name in your system.
The number of electrons for each atom is modified by its atomic
partial charge.

### Files

| option | filename | type | description |
| --- | --- | --- | --- |
| **-f** | traj.xtc | Input | Trajectory: xtc trr trj gro g96 pdb cpt |
| **-n** | index.ndx | Input, Opt. | Index file |
| **-s** | topol.tpr | Input | Run input file: tpr tpb tpa |
| **-ei** | electrons.dat | Input, Opt. | Generic data file |
| **-o** | density.xvg | Output | xvgr/xmgr file |

### Other options

| option | type | default | description |
| --- | --- | --- | --- |
| **-[no]h** | bool | no | Print help info and quit || **-[no]version** | bool | no | Print version info and quit || **-nice** | int | 19 | Set the nicelevel || **-b** | time | 0 | First frame (ps) to read from trajectory || **-e** | time | 0 | Last frame (ps) to read from trajectory || **-dt** | time | 0 | Only use frame when t MOD dt = first time (ps) || **-[no]w** | bool | no | View output .xvg, .xpm, .eps and .pdb files || **-xvg** | enum | xmgrace | xvg plot formatting: xmgrace, xmgr or none || **-d** | string | Z | Take the normal on the membrane in direction X, Y or Z. || **-sl** | int | 50 | Divide the box in this number of slices. || **-dens** | enum | mass | Density: mass, number, charge or electron || **-ng** | int | 1 | Number of groups of which to compute densities. || **-[no]symm** | bool | no | Symmetrize the density along the axis, with respect to the center. Useful for bilayers. || **-[no]center** | bool | no | Shift the center of mass along the axis to zero. This means if your axis is Z and your box is bX, bY, bZ, the center of mass will be at bX/2, bY/2, 0. |

### Known problems

- When calculating electron densities, atomnames are used instead of types. This is bad.

---

http://www.gromacs.org  
gromacs@gromacs.org
